# Application of Principal Component Analysis as a Prediction Model for Feline Sporotrichosis

**DOI:** 10.3390/vetsci12010032

**Published:** 2025-01-09

**Authors:** Franco Bresolin Pegoraro, Rita Maria Venâncio Mangrich-Rocha, Saulo Henrique Weber, Marconi Rodrigues de Farias, Elizabeth Moreira dos Santos Schmidt

**Affiliations:** 1 School of Veterinary Medicine and Animal Science (FMVZ), São Paulo State University (UNESP), Campus Botucatu, São Paulo 18618-687, Brazil; franco.pegoraro@unesp.br; 2 School of Medicine and Life Sciences, Pontifícia Universidade Católica do Paraná (PUCPR), Curitiba 80215-901, PR, Brazil; rita.rocha@pucpr.br; 3 Graduate Program in Animal Science, School of Medicine and Life Sciences, Pontifícia Universidade Católica do Paraná (PUCPR), Curitiba 80215-901, PR, Brazil; saulo.weber@pucpr.br (S.H.W.); marconi.farias@pucpr.br (M.R.d.F.)

**Keywords:** *Sporothrix* spp., cats, predictive function, fungus, plasma proteins

## Abstract

Sporotrichosis is a worldwide zoonotic disease that is spreading and causing epidemics in large urban centers. Cats are the most susceptible species to develop the disease, which could cause significant systemic lesions. The aim was to investigate and to identify predictive indicators of disease progression by correlations between the blood profile (hematological and biochemical analytes) and cutaneous lesion patterns of 70 cats diagnosed with *Sporothrix brasiliensis*. The higher occurrence in male cats in this study could be related to being non-neutered and having access to open spaces. Principal component analysis (PCA) with two principal components, followed by binary logistic regression, and binary logistic regression analysis, with independent variables and backward elimination modeling, were performed to evaluate hematological (n = 56) and biochemical (n = 34) analytes, including red blood cells, hemoglobin, hematocrit, leukocytes, segmented neutrophils, band neutrophils, eosinophils, lymphocytes, monocytes, total plasma protein, albumin, urea, creatinine, and alanine aminotransferase. Two logistic regression models (PCA and independent variables) were employed to search for a predicted model to correlate fixed (isolated) and disseminated cutaneous lesion patterns. Total plasma protein concentration may be assessed during screening diagnosis as it has been recognized as an independent predictor for the dissemination of cutaneous lesion patterns, with the capability of serving as a predictive biomarker to identify the progression of cutaneous lesions induced by *S. brasiliensis* infections in cats.

## Simple Summary

Sporotrichosis is a global zoonotic disease caused by the fungus *Sporothrix* spp. This fungus has been causing epidemics in urban centers, mainly in Latin America, with cats being the most susceptible species, serving as major vectors for disease transmission to humans. Our study investigated the predictive indicators of disease progression by examining the blood profiles and cutaneous lesions patterns of 70 cats diagnosed with *Sporothrix brasiliensis*. The results of this study were obtained by specific statistical analysis that correlated the blood profile of diseased cats with two cutaneous lesions patterns (fixed and disseminated). Of the 70 cats with confirmed sporotrichosis, 56 were males, a correlation that is closely linked to the animals’ behaviors of engaging in fighting, scratching trees, and roaming in open areas. In terms of blood profile, total plasma protein level was recognized as a predictive indicator of the progression of cutaneous lesion patterns in cats with this fungal infection.

## 1. Introduction

Sporotrichosis is a globally disseminated fungal zoonosis caused by *Sporothrix* spp. Over 50 species have been identified, with *S. schenckii*, *S. brasiliensis*, *S. globosa*, and *S. luriei* being the most frequent agents of cutaneous infections in humans and animal species, mainly in cats [1,2]. The infection is prevalent in tropical and subtropical regions, and it is considered to be the most frequent implantation mycoses in Latin America [3]. Brazil reported the highest number of globally diagnosed cases in cats and humans, with the southeastern region recognized as the epicenter of cat-transmitted sporotrichosis in humans [3,4,5]. More than 90% of urban sporotrichosis outbreaks are linked to *S. brasiliensis*, identified as the species with the highest pathogenicity index [6,7,8].

Cats are susceptible to infection and to disease transmission [3,8]. Species-specific behaviors, such as contact with soil and organic matter, territorial disputes, scratching, and biting, are the main sources of fungal propagation, with traumatic inoculation being the most common route of infection development [4,7,8,9,10,11]. The spread of sporotrichosis is significantly influenced by socioeconomic vulnerability and irresponsible pet ownership, as many infected cats are either semi-domesticated or stray animals [3,7,8,9,10,11].

Studies regarding the immune response in cats infected with *Sporothrix* are still limited. Cats generally develop poor granuloma formation and elevated fungal burdens in lesions, suggesting an inefficient immune response [1,10,11,12,13,14].

The incubation period for *Sporothrix* spp. in yeast form is approximately 14 days [3,11]. The initial lesions are firm, nodular, and isolated skin formations but tend to disseminate over time, progressing to ulcerations, crust formations, and granulomatous conditions [4,11,14]. The infection affects the skin, subcutaneous tissues, and lymphatic system, with the potential to disseminate to other organs, resulting in systemic disease. The clinical manifestations of sporotrichosis include cutaneous (isolated/fixed or disseminated with or without respiratory signs), lymphocutaneous, and extracutaneous forms, with the possibility of multiple presentations in the same animal [10,11,12,13,14,15]. There could be hematological and biochemical changes that could be related to an inefficient immune response during *Sporothrix* spp. infection in cats [1,4,10]. The diagnosis of feline sporotrichosis is based on the patient’s epidemiological profile, anamnesis, and clinical examination, including diagnostic tests such as fungal culture and lesion cytopathology [16]. Moreover, histopathology, immunohistochemistry, and molecular techniques can also be employed in clinical practice [5,14,15].

We hypothesized that hematological and biochemical analytes could be useful as predictive markers of the development of cutaneous lesions in feline sporotrichosis. Therefore, the aim of this study was to evaluate these analytes as potential predictors of lesion pattern differentiation by applying the principal component analysis method to identify possible correlations between analytes that could have an effect on the prediction of infection.

## 2. Materials and Methods

### 2.1. Ethical Statement

This study was approved by the Ethics Committee for Animal Use (CEUA/PUCPR n°8167130623), Curitiba, Brazil, and conducted in accordance with national legislation on animal protection.

### 2.2. Animals

This is a retrospective study. Clinical records and laboratory data were collected from the database of the Veterinary Teaching Hospital of PUCPR from August 2016 to August 2023. Four hundred and forty-eight patients included in this study were client-owned clinical cases diagnosed with sporotrichosis. Of these cats, 292 had laboratory results. However, complete clinicopathological data were available for only 75 cats. Five animals were excluded for concurrent diseases. Thus, a total of 70 cats diagnosed with sporotrichosis were included in this study. Clinical and laboratory data were extracted, including hematological and biochemical analytes, information regarding the sex of the animal, and the type of lesion was assessed for fixed cutaneous or disseminated cutaneous.

### 2.3. Laboratory Analysis

**Figure 1:**
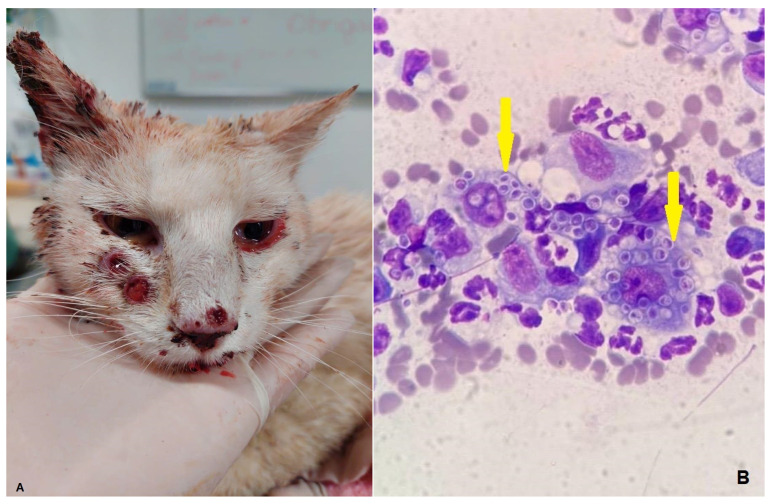
(**A**) Ulcerated cutaneous lesions in the face of a cat with *Sporothrix* spp. infection. (**B**) Cytological imprint (Diff Quick stain) of a *Sporothrix* spp. cutaneous lesion in a cat. Note the several yeast-like forms within macrophages (arrows).

The diagnosis of *Sporothrix* spp. infection in all cats (Figure 1A,B) was confirmed through cytological imprints of lesions in slides and microscopic visualization of the yeast by Diff Quick staining (Instant Prov Kit; Newprov^®^, Pinhais, Brazil) and by fungal culture of lesion exudates using a sterile swab. Samples were seeded onto Sabouraud dextrose 4% agar, supplemented with chloramphenicol and mycosel agar, and incubated at 25 °C. *Sporothrix* spp. was confirmed upon observed characteristic colony growth, which initially appears moist and white, by microscopic evaluation (×1000 magnification, Eclipse E200; Nikon^®^, Kobe, Japan) and by a lactophenol blue-stained smear to identify the characteristic hyphae and “daisy-like” clusters of *Sporothrix* spp. conidia [17,18].

Genotyping for *Sporothrix* spp. was conducted in 58 of the 70 cats. Different strains were used as a reference. *S. brasiliensis* 5110 (American Type Culture Collection MYA-4823) and *S. schenckii* CBS 130,112 were used as controls for molecular identification. *Sporothrix schenckii* CBS 130,112 and *S. schenckii* CBS 130,113 were used as controls. For amplified fragment length polymorphism (AFLP) fingerprinting, *S. schenckii* CBS 130099, *S. schenckii* CBS 130113, *S. schenckii* CBS 130114, *S. globosa* CBS 130116, *S. globosa* CBS 130,117, and *S. mexicana* CBS 132,926 were included. DNA extraction was performed using MagNA Pure 96 DNA, following the Pathogen 200 SV protocol (Roche Diagnostics, Roche Diagnostics GmbH), as previously described [19]. Briefly, a small portion of each culture was placed in a microtube with lysis solution (400 μL MagNA Pure Bacteria Lysis Buffer) and ceramic beads (MagNA Lyser Green Beads), after which the cells were lysed by a MagNA Lyser instrument (Roche Diagnostics) for 30 s at 6500 rpm, and the DNA was extracted and purified with MagNA Pure 96. A polymerase chain reaction (PCR) amplifying introns two to four and exons two to five of the calmodulin gene was used for species identification using primers Cmd5 5′-CCGAGTACAAGGARGCCTTC-3′ and Cmd6 5′-CCGATRGAGGTCATRACGTGG-3′, as previously described [20]. Amplicons were purified according to the AmpliClean method (NimaGen), and sequencing PCR was performed using 0.5 μL Brilliant Dye premix, 1.75 μL Brilliant Dye 5x sequencing buffer (NimaGen), 1 μL Cmd6 primer (5.0 μM), 5.75 μL water, and 1 μL purified DNA. Afterward, products were purified using the D-Pure purification protocol (NimaGen) and sequenced on a 3500 XL genetic analyzer (Applied Biosystems). The resulting calmodulin sequences were compared with NCBI GenBank sequences using BLAST (http://www.ncbi.nlm.nih.gov/, accessed on 1 June 2022) to determine their identity. The generated calmodulin sequences were deposited under GenBank accession numbers OQ571231-OQ571318.

*Sporothrix* isolates were subjected to amplified fragment length polymorphism (AFLP) genotyping using a method described previously [21]. In short, extracted DNA was submitted to a combined restriction-ligation using 2 U of EcoRI (New England Biolabs), 2 U of MseI (New England Biolabs), 50 pmol of EcoRI adapter (5′-TCGTAGACTGC GTACC-3′ and 5′-AATTGGTACGCAGTC-3′), 50 pmol of MseI adapter (5′-GACGATGAGTCCTGAC-3′ and 5′-TAGTCAGGACTCAT-3′), and 1 U of T4 DNA ligase (Promega). The restriction-ligation products were used in an amplification reaction with EcoRI (5′-FLU-GACTG CGTACCAATTCAC-3′) and MseI (5′-GATGAGTCCTGACTAAA-3′)-based primers, and amplicons were diluted 50× using water. A mix containing 1 μL of the diluted amplicons, 8.9 μL water, and 0.12 μL LIZ600 (Applied Biosystems) was submitted to a heating step at 95 °C for 1 min followed by 4 °C for 5 min and run on the ABI 3500XL genetic analyzer (Applied Biosystems), according to the manufacturer’s instructions. Data were analyzed using BioNumerics version 7.5 (Applied Maths, Sint-Martens-Latem, Belgium) with Pearson’s correlation coefficient and the unweighted pair group method with an arithmetic mean clustering algorithm. Genomic libraries were prepared and sequenced with the MiSeq platform (Illumina, San Diego, CA, USA) in 2 by 150-bp paired-end-read mode at Eurofins. Reads are publicly available at NCBI under BioProject ID: PRJNA836433. The read data were uploaded to the Galaxy tool, FastQC was used to assess read data quality, and no trimming was performed [22]. Sequenced strains were aligned against the *S. brasiliensis* 5110 reference genome (GCA_000820605.1) using BWA-MEM v0.7.17.1 [23]. Read duplicates were removed using RmDup, local realignment was performed using BamLeftAlign, and unpaired reads were removed with BAM Filter. Reads with a MAPQ score <60 were removed. Potential variations in genes associated with antifungal resistance were analyzed by visualization with JBrowse v1.16.11 [24].

The blood profile results from the clinical pathology laboratory data included, as hematological analytes, red blood cell count (RBC); hemoglobin; hematocrit (PCV); platelet counting; white blood cell count (WBC); and differential leukocyte count, including segmented neutrophils, band neutrophils, lymphocytes, monocytes, eosinophils, and basophils; concentrations of total plasma and serum protein, albumin, creatinine, and urea; and the serum activities of alanine aminotransferase (ALT) and gamma-glutamyl transferase (GGT), as biochemical analytes. In brief, complete blood counts (RBC and WBC) and hemoglobin concentrations were determined for every animal using an automated hematology analyzer (Ebram 18 Hemascreen; Ebram, São Paulo, Brazil). Hematocrit was determined by the microhematocrit method. Total plasma protein concentration was determined by refractometry. Total serum protein, albumin, creatinine, urea, ALT, and GGT were measured using an automated analyzer (Flexor EL80; EliTech Clinical Systems^®^, Puteaux, France), following the instructions of the manufacturer, using commercial kits (EliTech Clinical Systems^®^; Puteaux, France).

### 2.4. Statistical Analysis

The study focused on two main clinical patterns presentations of feline sporotrichosis: fixed cutaneous (G1) and disseminated cutaneous (G2). Normal distribution was tested using the Shapiro–Wilk normality test. The nonparametric Mann–Whitney test was used to compare the groups. Data are reported as median, percentiles (25 and 75%), and range (minimum and maximum), unless otherwise stated. These statistics were performed using the statistical software Graph-Pad Prism Version 9.5.1 for Windows (GraphPad Software, San Diego, CA, USA).

A cross-sectional observation study was conducted, and principal component analysis (PCA) was applied to evaluate associations between analytes and to reduce dimensionality. The components were established and subsequently organized to predict the lesion types of feline sporotrichosis, aiming to identify analytes related to disease progression. Additionally, independent variables were analyzed within the predictive model. The development of the predictive model for lesion patterns was based on hematological (n = 56) and biochemical (n = 34) data. Binary logistic regression was employed for each dataset using two methodologies: (1) PCA to reduce dimensionality, resulting in two principal components (PC1 and PC2), followed by binary logistic regression; and (2) binary logistic regression incorporating all independent variables with a backward elimination approach. For these analyses, PCA was presented with correlation matrices, eigenvalues, and eigenvectors, applied to assess the variance explained and convert variables into components in the logistic regression. The Kaiser–Meyer–Olkin (KMO) test and Bartlett’s test of sphericity were performed to assess the adequacy of the factor analysis. Moreover, a rotated component matrix using Varimax rotation and Kaiser normalization was generated to standardize coefficients and organize principal components. The Hosmer–Lemeshow test was implemented to evaluate the alignment of the logistic regression models. In the backward elimination model, variables with *p*-values > 0.05 (starting with the highest *p*-value) were manually removed from the model, reducing the number of predictors and including only significant variables. Principal component analyses and binary logistic regression statistics were performed using IBM^®^ SPSS Statistics Version 25 (IBM Corp., Armonk, NY, USA). For all analyses, *p*-values less than 0.05 were considered significant.

Outliers: To ensure accurate data analysis and in accordance with guidelines [25], values unrelated to the underlying disease (sporotrichosis) were excluded from the data distribution. Additionally, given the high incidence of platelet clumps and the limited number of total serum protein results, these analytes were excluded from PCA analysis. The final statistical analysis included the following analytes: RBC, hemoglobin, PCV, WBC, segmented neutrophils, band neutrophils, lymphocytes, eosinophils, monocytes, total plasma protein, albumin, creatinine, urea, and ALT.

## 3. Results

Of the 70 cats with confirmed sporotrichosis diagnosis and complete clinicopathological data, 56 were males (80%) and 14 females (20%). All 58 cats had positive genotyping (identification and isolation) results for *Sporothrix brasiliensis*, independently of the type of lesion and/or sex. Cats were separated into two groups based on the type of lesions: Group 1 (G1) fixed cutaneous (n = 13; 18.5%): 12 males and one female; Group 2 (G2) disseminated cutaneous (n = 57; 81.5%): 47 males and 10 females. Of the cats with fixed cutaneous lesions, nine males and one female were non-neutered, three were neutered, and the information was not available for one cat. Additionally, seven domestic males and the female in G1 had access to open spaces, and four males did not have access to open spaces. Regarding cats with disseminated cutaneous lesions (G2), 31 males and 6 females were non-neutered, 14 males and 4 females were neutered, and the information was not available for 3 animals. Of the cats in G2, 31 domestic males and 4 females had access to open spaces; 3 males and 4 females did not have access to open spaces, and 8 males and 2 females were kept outside all the time (half-domestic).

The results for the hematological analyte comparison of both groups are presented in Table 1. Cats in G2 had significantly higher segmented neutrophils and total plasma protein when compared to those in G1. Red blood cells, hematocrit, hemoglobin, WBC, band neutrophils, lymphocytes, eosinophils, monocytes, and platelets counts were not significantly different between groups and were within RI [26] (Table 1).

The biochemical analyte comparison results for G1 and G2 are presented in Table 2. No significant differences were found for albumin, creatinine, urea, the activities of GGT and ALT, and total serum protein between G1 and G2. However, albumin concentrations were below the RI [27] for both groups of cats.

**Figure 2:**
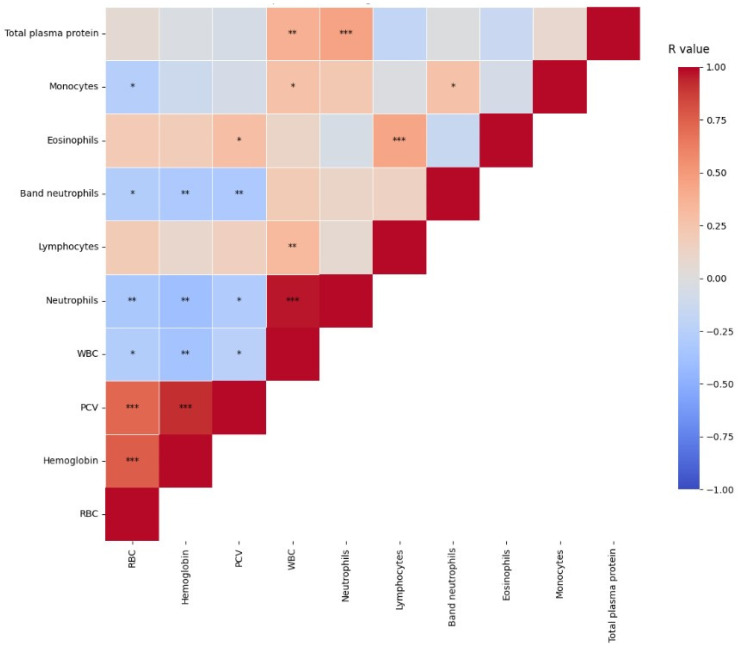
Heatmap for hematological analyte correlations of cats with sporotrichosis. Ranges to positive correlations as dark red (r = 1) and negative correlations as dark blue (r = −1). *p*-values of significance are indicated as *, *p* < 0.05, **, *p* < 0.01, and ***, *p* < 0.001. (PCV—hematocrit; neutrophils—segmented neutrophils; r value—correlation coefficient).

The correlations among the ten hematological analytes obtained by the PCA model are presented in Figure 2. Significant positive correlations were observed for PCV with RBC (r = 0.7, *p* < 0.001) and hemoglobin (r = 0.9, *p* < 0.001). Red blood cells with hemoglobin (r = 0.7, *p* < 0.001) and segmented neutrophils with WBC (r = 0.9, *p* < 0.001) were also positively correlated. Additionally, segmented neutrophils with TPP and lymphocytes with eosinophils had significantly positive moderated correlations (r = 0.4, *p* < 0.001).

Low negative correlations were observed between hemoglobin, WBC, and segmented and band neutrophils (r = 0.3, *p* < 0.01). Although there were other statistically significant (*p* < 0.05) correlations, they were weak (r = 0.2) (Figure 2). Significant correlations were not observed between urea, creatinine, GGT, and ALT (Figure S1).

For the hematological analyte evaluation performed by PCA, the first (PC1) and second principal (PC2) rotated components explained 95.2% and 4.1% of the variance in the dataset, respectively. Together, PC1 and PC2 captured 99.3% of the total variability, indicating that these components effectively summarized the dataset (Table S1). For the biochemical PCA analysis, PC1 rotated components explained 90.0% of the variance. When both components were considered, they accounted for 99.9% of the total variability (Table S2).

**Figure 3:**
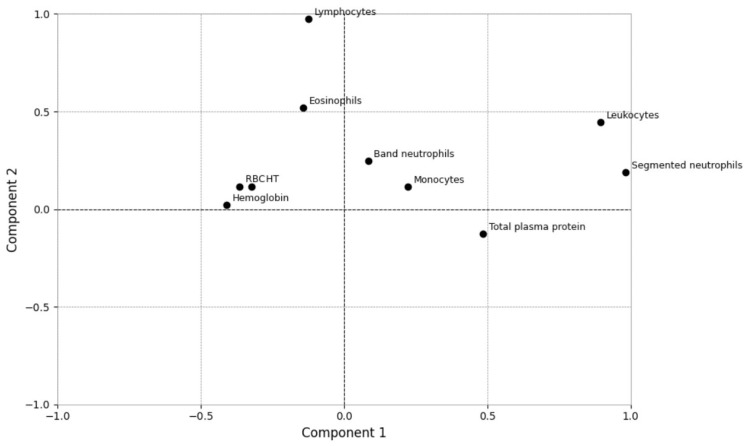
Biplot representation of hematological analytes of cats with sporotrichosis and its dispersion. (PCV—hematocrit; leukocytes—WBC; neutrophils—segmented neutrophils).

**Figure 4:**
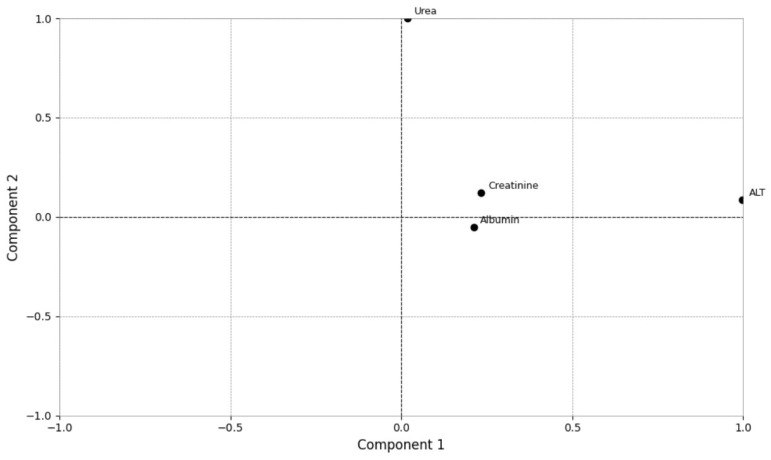
Biplot representation of biochemical analytes in cats with sporotrichosis and its dispersion. (ALT—alanine aminotransferase).

For the hematological loading (i.e., biplot) analysis, clustering was observed among RBC, PCV, and hemoglobin, indicating a strong correlation in terms of principal components. Total WBC, segmented and band neutrophils, monocytes, and TPP were correlated with Component 1 (horizontal axis). Lymphocyte had the highest loading on Component 2 (vertical axis) (Figure 3). Regarding the biochemical biplot, urea had a strong correlation with Component 2, while ALT had a strong correlation with Component 1. Albumin and creatinine were closer positioned, indicating their similar contribution with Component 1, but with a weak correlation (Figure 4).

The predictive model of lesion type in feline sporotrichosis based on the principal components (PC1 and PC2) of hematological analytes explained 80.4% of the analyzed cases (cutoff value: 0.500). For biochemical analytes, the model explained 88.2% of cases (cutoff value: 0.500) (Tables S3 and S4).

The logistic regression results had a significant level (*p* = 0.04) for hematological PC1 when compared to lesion pattern, but the predictor had no effect on the odds ratio (OR) (OR = 1.0). Neither PC2 nor the constant were statistically significant in this case (Table S5). For the biochemical principal components, neither PC1, PC2, nor the constant were statistically significant (Table S6). The logistic regression analysis developed for a predictive model associated with the most frequent lesion patterns observed in cats diagnosed with sporotrichosis (G1 and G2), the proposed model between independent hematological and biochemical analytes, correctly classified 76.8% of the independent hematological cases, and for the biochemical analytes, the rate was 88.2% (cutoff value for both analyses = 0.5) (Tables S7 and S8).

Backward elimination begins by fitting a multiple linear regression model with all independent variables (Table S9). The analytes with the highest *p*-values are removed from the model and a new model is produced. This process is repeated until the analyte has a *p*-value below the threshold, typically *p* = 0.05. The analytes that do not reach the threshold are removed. In this study, TPP was the unique statistically significant and constant result for an analyte: *p* = 0.01 and *p* = 0.04, respectively (Table 3). By this analytical method, an odds ratio of 3.3-fold increase for TPP was observed. Thus, the predictive function calculated from our results meant that, for each increase of 1 g/dL (or for every 10 g/L) in TPP concentration in cats with sporotrichosis, the chance of developing disseminated cutaneous lesions was increased 3.3-fold. Regarding the biochemical analytes (Table 3), the only remaining significant analyte was urea (*p* = 0); however, the predictor had no effect on the odds ratio (OR = 1.0).

Following these results, a predictive function was developed based on the evidence of TPP obtained in the binary logistic regression analysis demonstrating the possibility of progression from a fixed cutaneous lesion to a disseminated cutaneous pattern. The formula for calculating this predictive function is expressed as follows:

$$P=\frac{1}{1+e^{-(-7.789+1.201\times total\;plasma\;protein)}}$$

## 4. Discussion

Consistent with previous reports, most of the animals in this study were male cats with disseminated cutaneous lesions [12,14,28,29,30]. The higher occurrence found in domestic males of this study could be related to having access to open spaces and being non-neutered, which could include the species’ habits of fighting and scratching trees [3,16]. Furthermore, there is a prevailing presence of non-neutered male cats that are infected with *Sporothrix* spp. [3,12,15,28,29,30].

Previous investigations in humans and Murinae suggested that both innate and adaptative immune response could be similar to the anti-*Sporothrix* response in cats [10,11,31,32,33]. Nevertheless, the immune response to feline sporotrichosis is still not well understood [28]. It is known that cats infected with *S. brasiliensis* or other virulent *Sporothrix* species have the potential to develop disseminated infections, suggesting the ability of the fungus to interfere with Th1-mediated immunity [4,8,11,16,34]. Thus, cell-mediated immunity appears to play a key role in the antifungal response in *Sporothrix* spp. infection in cats, and the Th1 cellular response could be the specific tool for controlling the disease by aiding in the growth of granulomas [10,11,30,31,34]. Additionally, in other fungal infections like histoplasmosis, candidiasis, and cryptococcosis, animals with a compromised Th1 immune response are susceptible to more severe and disseminated infection [35,36,37].

The main changes observed in cats with *Sporothrix* spp. infection were significant increases in segmented neutrophils and TPP concentrations in cats with the disseminated cutaneous (G2) lesion pattern. These findings could reflect the response to concomitant fungus and bacterial infection, suggesting an intense neutrophil chemotaxis induced by cell-mediated immunity [10,34,38] and activation of the acute phase response, which increases the concentration of serum globulin [39]. In this study, Component 1 had a strong association with inflammatory response analytes (WBC, segmented neutrophils, and total plasma protein) (Figure 3) and the logistic regression analysis demonstrated that PC1 had a significant value (*p* = 0.04), suggesting a correlation between these analytes and worsening sporotrichosis skin lesions.

In murine models, the humoral response induced by Th2 cells induces antibody production, especially IgE and IgG [33,34,35], which was also suggested by an investigation in cats responding to *Sporothrix* antigens [40]. These increased Ig concentrations result in hyperproteinemia. Furthermore, cats from both groups of this study had hypoalbuminemia when compared to RI values. These findings, combined with segmented neutrophilia and high TTP concentrations, could indicate sustained production of immunoglobulins in response to sporotrichosis and to the systemic inflammation caused by disseminated cutaneous lesions [10,11,30,31,40]. Additionally, hypoalbuminemia associated with hyperglobulinemia was previously found in *Sporothrix* spp. infections in cats [15]. It is worth mentioning that the cats in this study did not have clinical or laboratory signs of dehydration.

In veterinary medicine, there are low numbers of PCA and predictive model studies about blood analytes. In dogs with mammary tumors, the combined PCA of leukogram, albumin/globulin, age, and tumor size was a strong predictor of survival rate [41]. In addition, a non-effusive feline infectious peritonitis predictive diagnosis was also developed with PCA analysis [42]. Our study evaluated two basic logistic regression models (PCA and independent variables) for hematological and biochemical analytes in cats with sporotrichosis. Both performed well and successfully identified that the TPP concentration could be considered as a predictive marker for cutaneous lesions in cats with sporotrichosis. Therefore, the evidence of TPP obtained by the binary logistic regression analysis demonstrates the possibility of progression from a fixed cutaneous lesion to a disseminated cutaneous pattern. Our hypothesis is that increased TPP concentration is strongly associated with a probability of the development of disseminated cutaneous lesions in feline sporotrichosis, suggesting its potential as a future prognostic prospect.

## 5. Conclusions

In conclusion, the changes found in this study could provide an overview of the hematological and biochemical effects produced by *S. brasiliensis* infection in cats with fixed and disseminated cutaneous lesions. Total plasma protein concentration could be a valuable marker for identifying cats at risk of developing more severe disseminated cutaneous lesions and the calculated predictive function could be considered as a screening tool for cats with sporotrichosis. Total plasma protein determination during daily routine laboratory evaluations could have the potential of serving as a predictive biomarker to identify the progression of cutaneous lesions induced by *S. brasiliensis*, especially in an endemic area for this infection.

## Figures and Tables

**Table 1:** Results of the comparison of hematological analytes between cats with sporotrichosis: median, range (minimum and maximum), interquartile [25% and 75%], *p*-value, and reference interval (RI).

Analytes	Group 1 (n = 13) Median (Min–Max) [25–75%]	Group 2 (n = 57) Median (Min–Max) [25–75%]	*p* Value	RI [20]
RBC (10^6^/µL)	7.87 (5.08–9.88) [6.68–9.45]	7.42 (3.65–12.3) [6.10–8.86]	0.370	5–10
Hemoglobin (g/L)	127 (100–166) [109–140]	117 (64–175) [89–131]	0.063	80–150
Hematocrit (%)	40 (28–45) [32.5–41.7]	35.5 (19–48) [29.7–40]	0.149	30–45
WBC (/µL)	11,150 (6100–22,600) [7288–15,638]	14,675 (4250–46,200) [9825–20,975]	0.128	5500–19,500
Segmented Neutrophils (/µL)	5931 (2869–13,728) [3616–9042]	9923 (2805–44,352) [5319–12,360]	0.027	2500–12,500
Band neutrophils (/µL)	30.5 (0–624) [0–146.3]	65 (0–3048) [0–541]	0.329	0–300
Lymphocytes (/µL)	3445 (1761–9270) [2372–4837]	3,194 (408–9168) [1775–4842]	0.347	1500–70,000
Eosinophils (/µL)	723 (176–1390) [543–1081]	433 (0–3850) [216–733]	0.032	100–1500
Monocytes (/µL)	228 (0–1408) [61.7–626.3]	395.5 (0–1899) [133–875]	0.247	100–1400
Platelets (10^3^/µL)	300 (240–516) [249–401]	337 (195–860) [283–459]	0.24	300–800
Total plasma protein (g/L)	71 (64–82) [65–79]	78 (62–120) [74–84]	0.019	60–80

G1: Fixed cutaneous; G2: disseminated cutaneous. Mann–Whitney U tests. *p*-values less than 0.05 are considered statistically significant. RBC: total red blood cells count; WBC: total white blood cell count.

**Table 2:** Results of the comparison of biochemical analytes between cats with sporotrichosis: median, range (minimum and maximum), interquartile [25% and 75%], *p*-value, and reference interval (RI).

Analytes	Group 1 (n = 13) Median (Min–Max) [25–75%]	Group 2 (n = 57) Median (Min–Max) [25–75%]	*p* Value	RI [21]
Total serum protein * (g/L)	75 (63–76) [63–76]	76 (62–86) [67–81]	0.558	60–80
Albumin (g/L)	26.9 (15.2–31.5) [16.9–30.6]	26 (9.1–33.9) [18.11–28.7]	0.532	28–39
Creatinine (µmol/L)	116.6 (99–135.2) [107.8–121.1]	102.5 (30.9–164.4) [86.6–121.1]	0.053	70–177
Urea (mmol/L)	8.79 (6.62–11.2) [7.72–10.4]	7.70 (1.64–16.5) [6.82–9.27]	0.171	3.6–10.7
ALT (U/L)	54.2 (5.0–79.2) [30.0–74.0]	29.6 (1.3–216.0) [11.7–57.0]	0.325	10–100
GGT (U/L)	2.0 (1.3–3.9) [1.5–2.8]	2.0 (0.7–9.6) [2–3.2]	0.267	0–6

G1: Fixed cutaneous; G2: disseminated cutaneous. Mann–Whitney U tests. *p*-values less than 0.05 are considered statistically significant; * n = 12 cats for each group.

**Table 3:** Binary logistic regression analysis with the final analytes (urea and total plasma protein) of cats with sporotrichosis obtained by manual backward elimination modeling for independent variables.

Analytes	B	S.E.	Wald	df	Sig.	Exp (B)	95% C.I. for EXP (B)	
							Inferior	Superior
Urea	0.037	0.011	12.190	1.000	0.000	1.038	1.016	1.060
Total plasma protein	1.201	0.509	5.562	1.000	0.018	3.322	1.225	9.012
Constant	7.789	3.786	4.234	1.000	0.040	0.000	-	-

Urea (*p* = 0), total plasma protein (*p* = 0.01) and constant (*p* = 0.04) were significant. (B: regression coefficient; S.E.: standard error; df: degrees of freedom; Sig.: significance; Exp (B): exponentiate coefficient (odds ratio); C.I.: confidence interval).

## Data Availability

The data presented in this study are available in the Supplementary Materials.

## Supplementary Materials

The following supporting information can be downloaded at: https://www.mdpi.com/article/10.3390/vetsci12010032/s1. Figure S1: Heatmap for biochemical analyte correlations of cats with sporotrichosis. Ranges to positive correlations as dark red (r = 1) and negative correlations as dark blue (r = −1). There was no statistically significant difference. *p* > 0.05 (ALT, alanine aminotransferase; r value—correlation coefficient). Table S1: Hematological analytes of cats with sporotrichosis with raw and rescaled eigenvalues of the two principal components (PC1 and PC2). Table S2: Biochemical analytes of cats with sporotrichosis with raw and rescaled eigenvalues of the two principal components (PC1 and PC2). Table S3: Binary classification and predictive model of principal component analysis for hematological analytes in cats with sporotrichosis. Table S4: Binary classification and predictive model of principal component analysis for biochemical analytes in cats with sporotrichosis. Table S5: Logistic regression equation for hematological analytes of cats with sporotrichosis (PC1 and PC2 variables). Table S6: Logistic regression equation for biochemical analytes of cats with sporotrichosis (PC1 and PC2 variables). Table S7: Binary classification for hematological analytes of cats with sporotrichosis. Table S8: Binary classification for biochemical analytes of cats with sporotrichosis. Table S9: Backward elimination model equation with independent hematological analytes of cats with sporotrichosis.
