# Supplementary material for: Application of Principal Component Analysis as a Prediction Model for Feline Sporotrichosis
Source: Vet Sci. 2025 Jan 9;12(1):32. doi: 10.3390/vetsci12010032 (PMC11768719; doi:10.3390/vetsci12010032)
Supplement: Supplementary file 1 [file vetsci-12-00032-s001.zip › vetsci-3333306-supplementary.pdf]

Supplementary Materials

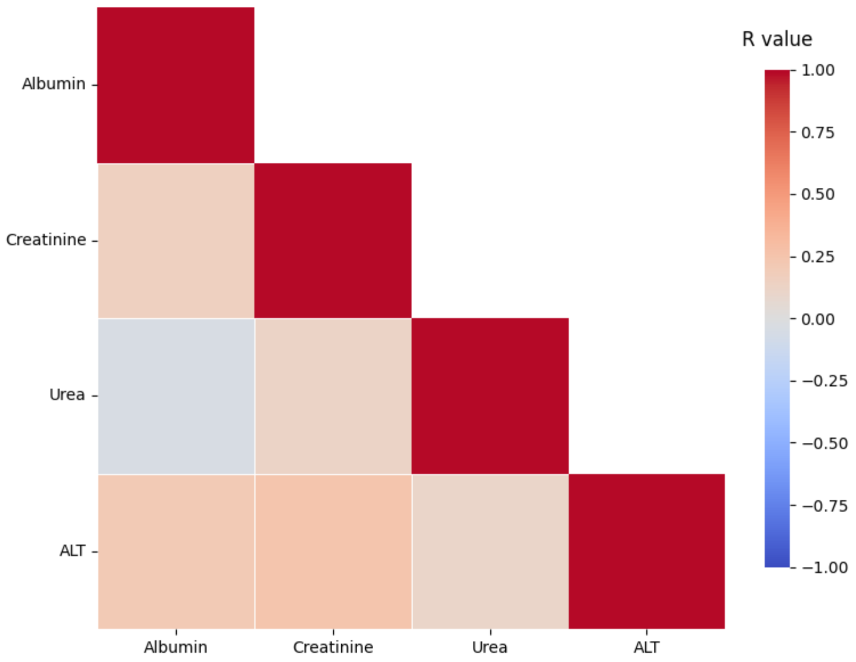

Figure S1: Heat map for biochemical analytes correlations of cats with sporotrichosis. Ranges to positive as dark red (r = 1) and negative correlation as dark blue (r = -1). There was no statistically significant difference. $P > 0.05$ (ALT, alanine aminotransferase; r value - correlation coefficient).

Table S1. Hematological analytes of cats with sporotrichosis with raw and rescaled eigenvalues of the two principal components (PC1 and PC2).

Component		Sum of Squared Loadings (Extraction)			Sum of Squared Loadings (Rotation)		
		Total	% Variance	% Cumulative	Total	% Variance	% Cumulative
Raw	1	176914164.2	95.182	95.182	157954338.8	84.982	84.982
	2	7674629.558	4.129	99.311	26634455.03	14.33	99.311
Rescaled	1	2.571	25.711	25.711	2.495	24.947	24.947
	2	1.492	14.92	40.631	1.568	15.684	40.631

PC1 and PC2 together captured 99.3% of total variability

Table S2: Biochemical analytes of cats with sporotrichosis with raw and rescaled eigenvalues of the two principal components (PC1 and PC2).

Component		Sum of Squared Loadings (Extraction)			Sum of Squared Loadings (Rotation)		
		Total	(%) Variance	(%) Cumulative	Total	(%) Variance	(%) Cumulative
Raw	1	1634.465	90.088	90.088	1619.999	89.291	89.291
	2	179.468	9.892	99.98	193.934	10.689	99.98
Rescaled	1	1.115	27.881	27.881	1.092	27.297	27.297
	2	1.001	25.017	52.898	1.024	25.602	52.898

Both components accounted for 99.9% of total variability

Table S3. Binary classification and predictive model of Principal Component Analysis for hematological analytes in cats with sporotrichosis

		Predicted Outcome		Percent correct
**Binary Classification Table**		**F**	**D**	
	**F**	1	11	8.3
**Observed Outcome**	**D**	0	44	100
**Percentage global**				80.4

(F) fixed and (D) disseminated cutaneous lesions

Table S4. Binary classification and predictive model of Principal Component Analysis for biochemical analytes in cats with sporotrichosis

		Predicted Outcome		Percent correct
**Binary Classification Table**		**F**	**D**	
	**F**	0	4	0
**Observed Outcome**	**D**	0	30	100
**Percentage global**				88.2

(F) fixed and (D) disseminated cutaneous lesions

Table S5. Logistic regression equation for hematological analytes of cats with sporotrichosis (PC1 and PC2 variables)

	B	S.E.	Wald	df	Sig.	Exp(B)	95% C.I. for EXP(B)	
							**Lower**	**Upper**
**PC1**	0	0	4.11	1	0.043	1	1	1
**PC2**	0	0	1.403	1	0.236	1	1	1
**Constant**	0.605	0.877	0.477	1	0.49	1.832		

PC1: P = 0.043. B: regression coefficient; S.E: standard error; df: degrees of freedom; Sig.: significance; Exp(B): exponentiated coefficient (odds ratio); C.I: confidence interval.

Table S6. Logistic regression equation for biochemical analytes of cats with sporotrichosis (PC1 and PC2 variables).

	B	S.E.	Wald	df	Sig.	Exp(B)	95% C.I. for EXP(B)	
							**Lower**	**Upper**
**PC1**	-0.003	0.013	0.048	1	0.827	0.997	0.972	1.023
**PC2**	-0.076	0.054	1.957	1	0.162	0.927	0.834	1.031
**Constant**	5.922	3.021	3.843	1	0.05	373.287		

All variables: no significant difference. B: regression coefficient; S.E: standard error; df: degrees of freedom; Sig.: significance; Exp(B): exponentiated coefficient (odds ratio); C.I: confidence interval.

Table S7. Binary classification of hematological analytes in cats with sporotrichosis

		Predicted Outcome		Percent correct
**Binary Classification Table**		**F**	**D**	
	**F**	3	9	25
**Observed Outcome**	**D**	4	40	90.9
**Percentage global**				76.8

(F) fixed and (D) disseminated cutaneous lesions

Table S8. Binary classification of biochemical analytes in cats with sporotrichosis

		Predicted Outcome		Percent correct
**Binary Classification Table**		**F**	**D**	
	**F**	0	4	0
**Observed Outcome**	**D**	0	30	100
**Percentage global**				88.2

(F) fixed and (D) disseminated cutaneous lesions

Table S9. Backward elimination model equation with independent hematological analytes of cats with sporotrichosis

Analytes	B	S.E.	Wald	df	Sig.	Exp(B)	95% C.I. for EXP(B)	
							Lower	Upper
**RBC**	0.09	0.395	0.052	1	0.82	1.094	0.504	2.374
**Hemoglobin**	-0.353	0.474	0.556	1	0.456	0.702	0.278	1.777
**Hematocrit**	0.046	0.171	0.071	1	0.789	1.047	0.749	1.463
**WBC**	0	0.004	0.005	1	0.946	1	0.992	1.008
**Segmented neutrophils**	0	0.004	0.009	1	0.926	1	0.992	1.009
**Lymphocytes**	0	0.004	0	1	0.987	1	0.992	1.008
**Band neutrophils**	0.002	0.004	0.195	1	0.659	1.002	0.994	1.01
**Eosinophils**	0	0.004	0.006	1	0.938	1	0.992	1.009
**Monocytes**	0	0.005	0.005	1	0.946	1	0.991	1.009
**Total plasma protein**	1.221	0.726	2.828	1	0.093	3.39	0.817	14.067
**Constant**	-6.246	5.214	1.435	1	0.231	0.002		

RBC: total red blood cells; WBC: total white blood cells; B: regression coefficient; S.E: standard error; df: degrees of freedom; Sig: significance; Exp(B): exponentiated coefficient (odds ratio); C.I: confidence interval.
